# Interaction and behaviour imaging: a novel method to measure mother–infant interaction using video 3D reconstruction

**DOI:** 10.1038/tp.2016.82

**Published:** 2016-05-24

**Authors:** C Leclère, M Avril, S Viaux-Savelon, N Bodeau, C Achard, S Missonnier, M Keren, R Feldman, M Chetouani, D Cohen

**Affiliations:** 1 Institut des Systèmes Intelligents et de Robotiques, CNRS, UMR 7222, Sorbonne Universités, Université Pierre et Marie Curie, Paris, France; 2 Department of Child and Adolescent Psychiatry, Pitié-Salpêtrière Hospital, AP-HP, 47-83, Boulevard de l'Hôpital, Paris, France; 3 Laboratoire de Psychologie Clinique et Psychopathologie, Psychanalyse, EA4056, Paris V René Descartes University, Avenue Paul Vaillant Couturier, Boulogne, France; 4 Department of Psychiatry, Infant Mental Health Unit, Geha Hospital, Tel Aviv University, Tel Aviv, Israel; 5 Department of Psychology and the Gonda Brain Sciences Center, Bar-Ilan University, Ramat-Gan, Israel

## Abstract

Studying early interaction is essential for understanding development and psychopathology. Automatic computational methods offer the possibility to analyse social signals and behaviours of several partners simultaneously and dynamically. Here, 20 dyads of mothers and their 13–36-month-old infants were videotaped during mother–infant interaction including 10 extremely high-risk and 10 low-risk dyads using two-dimensional (2D) and three-dimensional (3D) sensors. From 2D+3D data and 3D space reconstruction, we extracted individual parameters (quantity of movement and motion activity ratio for each partner) and dyadic parameters related to the dynamics of partners heads distance (contribution to heads distance), to the focus of mutual engagement (percentage of time spent face to face or oriented to the task) and to the dynamics of motion activity (synchrony ratio, overlap ratio, pause ratio). Features are compared with blind global rating of the interaction using the coding interactive behavior (CIB). We found that individual and dyadic parameters of 2D+3D motion features perfectly correlates with rated CIB maternal and dyadic composite scores. Support Vector Machine classification using all 2D–3D motion features classified 100% of the dyads in their group meaning that motion behaviours are sufficient to distinguish high-risk from low-risk dyads. The proposed method may present a promising, low-cost methodology that can uniquely use artificial technology to detect meaningful features of human interactions and may have several implications for studying dyadic behaviours in psychiatry. Combining both global rating scales and computerized methods may enable a continuum of time scale from a summary of entire interactions to second-by-second dynamics.

## Introduction

Parent–child interactions are crucial for the formation of attachment bonds, healthy development, learning and well-being, as well as later psychopathology.^1, 2^ In many species, including mammals, parent–child interactions are based on close relationships that are characterized by (i) infant dependency on caregivers and (ii) specific communication dynamics associated with caregiver's adaptation and infant maturation.^3^ Studying the quality and dynamics of early interactions is a complex endeavour as it requires the perception and integration of multimodal social signals and the understanding of how two interactive partners synchronize.^4, 5^ In human, behavioural/social signals include imitation and mimics,^6^ gazing,^7^ vocalization and speech turns,^8^ motion,^9^ motherese and emotional signals^10^ and interpersonal synchrony.^4^ Combining several approaches within a multidisciplinary perspective at the intersection of social signal processing, computational neuroscience, developmental psychology and child psychiatry may be useful for investigating the meaning of social signals during early parent–child interaction.^11, 12^ Furthermore, exploring normal and pathological interactions during this early period of life offers the possibility to detect distress signals that the infant or parent cannot express directly. Similarly, defining the neural^13^ and hormonal^14^ correlates of behaviourally synchronic interactions provide validation for the crucial value of studying synchrony during child development.^4^ It appears that synchrony should be regarded as a social signal *per se* as it has been shown to be valid in both normal and pathological populations. Better parent–child synchrony during interactions with both mother and father is associated with greater familiarity (vs unknown partner), healthy parenting (vs psychopathology), typical development (vs psychopathological development).^15^ Furthermore, several longitudinal studies, some spanning infancy to adolescence, demonstrate the positive effect of early parent–child synchrony on a host of positive outcomes, including empathy, emotion regulation, social competence, less internalizing and externalizing problems, and the capacity to engage in reciprocal dialogue with close friends.^16, 17, 18^

Automatic computational methods theoretically offer the possibility to extract and analyse communication of several partners simultaneously by taking an integrative perspective, considering the multimodal nature and dynamics of social signals/behaviours, and measuring synchrony between partners' actions.^19^ Few seminal studies tried to apply social signal processing to mother–infant interaction focusing on head movements,^20^ facial expression,^21^ motherese^22^ and speech turn.^1, 8^ In the era of RGB-D sensors (for example, Microsoft Kinect), new body movement cues have been proposed based on the online extraction of the skeleton.^23, 24^ We previously developed an original setup to understand the clinical relevance of dyadic interactions using two-dimensional plus three-dimensional (2D+3D) video sensors to monitor free play sessions of mother–infant interaction. We also defined several motion features (see below) based on a gaming task for studying mother–infant interaction and developed computational models to detect them in natural settings.^25^ The aim of the current study was to show the validity of the aforementioned automatic method by comparing our methods with the well-validated coding interactive behavior (CIB).^26^ To do this, we characterized early mother–infant interaction occurring in situations of severe emotional neglect and of typical development using both the CIB and our automatic measures of individual and dyadic motion features.^26^ To validate our methods, we performed correlation analyses of CIB composite scores and motion features, and machine learning classification based on motion features to predict group classification (control dyads vs dyads with mother showing neglect). The CIB is a global rating system for assessing social interactions with good psychometrics, including construct and predictive validity, test–retest reliability in repeated observations from infancy to adolescence, and associations with brain activations, hormonal patterns and physiological response. The system has been utilized across ages from new-born to adolescence, applied across multiple cultures and interactive partners (mothers, fathers, caregivers, strangers, friends and couples) and has proved useful in detecting differences related to parent or child age, interactive context (for example, play vs feeding), cultural variations, biological and social-emotional psychopathology, and change following intervention.^27^

## Materials and methods

### Participants

The protocol was approved by the Pitié-Salpétriêre hospital ethics committee (*Comité de Protection des Personnes*). All the participants received written and oral information on the experiment and gave written consent before participation. The participants were recruited in a French perinatal ambulatory unit ‘*Unité Petite Enfance et Parentalité Vivaldi*' of the Pitié-Salpêtrière University Hospital. Dyads (*N*=10) consisted of mothers with their children whose age varied between 12 and 36 months, referred to the unit by paediatricians, social services or court petitions due to child neglect. Emotional abuse and neglect is a common form of child maltreatment. However, emotional neglect is particularly less documented due to its insidious form.^28^ It refers to omission, unlike commission in abuse^29^ and remains less apparent than physical maltreatment. A conceptual framework has arrived at a working definition of emotional neglect as persistent, non-physical, harmful interactions with the child by the caregiver.^30^ Clinical confirmation of impaired caregiver–infant interaction was based on a child psychiatrist's assessment using the PIRGAS scale (Parent–Infant Relationship Global Assessment Scale, Axe II of DC 0–3 R), a clinical intensive scale of parent–child interaction quality. A control group of dyads with normal development and without interactional difficulty (*N*=10) was also recruited. Demographics and clinical characteristics of the dyads are given in Table 1. As we aimed to use correlation analysis, it was important to have a large distribution of PIRGAS scores between the two groups and within all dyads combined. This was the case since we found that PIRGAS scores between the two groups were significantly different (see Table 1) and that PIRGAS scores showed a large distribution with ranges from 25 to 92.

### Global rating of interaction

To assess the quality of early interaction during free play video sessions, we used the CIB,^26, 27^ which is one of the most often used and validated global interaction scales.^15^ The CIB is a global rating system of parent–child interaction that contains both micro-level codes and global rating scales. Each code is rated from 1 (a little) to 5 (a lot). Forty-three different codes are grouped into several interactive composites. Codes were averaged into composites that were theoretically derived, concerned with diverse aspects of early parent–infant relationships and showed acceptable to high levels of internal consistency.^27, 31^ The French version has been validated and offers the same factorial distribution.^32^ Eight composite scores were used in the current study focusing on the mother (*N*=3), the infant (*N*=3) and the dyad (*N*=2): Maternal sensitivity was the average of maternal acknowledgement of infant interactive signals, imitation and elaboration of the infant's behaviour, gaze directed to the infant or joint activity, appropriate tone of voice/motherese, expression of positive and appropriate range of affect, resourcefulness in dealing with infant negative states, affectionate touch, supportive presence and infant-led interaction (the degree to which interactions were judged to be led by the infant, due to parental focus on the child needs and states rather than their own). Mother intrusiveness was the average of maternal inappropriate physical manipulation, mother overriding behaviour (the degree to which mother disregards the infant's signals and interrupts the infant's ongoing behaviour), maternal negative affect/anger toward the baby, maternal anxiety, maternal criticizing of infant's behaviour and mother-led interaction (the degree to which interactions were judged to be led by the mother's needs rather than the infant's needs, pace and agenda). Mother limit setting was the average of consistency of parental style, resourcefulness and appropriate structure, limit setting. Dyadic reciprocity(synchrony) was the average of the mother's elaboration of the infant's vocalizations and movements, parental gaze directed to the infant, infant gaze directed to the parent or joint activity, verbal praises to the infant's behaviour, affectionate touch and enthusiasm, infant vocalization/verbal output, warm and positive affect for both mother and child, dyadic adaptation-regulation and fluency of the interaction. Negative dyadic status was the average of maternal negative affect/anger, the mother's hostility behaviour, the child negative and labile affect, withdrawal from the environment, interactive constriction and tension. Infant avoidance was the average of child negative and labile affect, withdrawal from the environment and avoidance behaviour toward the mother. Infant engagement was the average of joint attention, child positive affect, affection to parent, alertness, fatigue, vocalizations/verbal output, initiation, competent use of the environment, creative-symbolic play and infant-led interaction. Infant compliance was the average of compliance to parent, reliance on parent for help and on-task persistence. CIB composite scores were given after viewing the whole 4-min sequence of interaction.

### Setting and automatic extraction of individual and dyadic motion features

**Figure 1:**
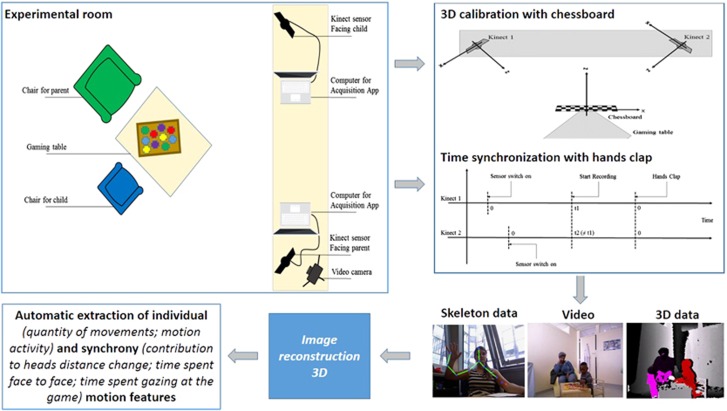
Experimental setup for capture and extraction of two-dimensional plus three-dimensional (2D+3D) motion feature during mother–infant early interaction. Two Kinect cameras (one for the mother and one for the infant) record the interaction. To permit 3D reconstruction, the video data are synchronized for time through hand clapping and spatial 3D calibration is possible through a chessboard used at the beginning of the recording. The 2D images, 3D images and skeleton are recorded during infant–mother interaction. After the 3D reconstruction, several 2D+3D motion features are extracted at both individual (quantity of movements, motion activity) and synchrony levels (contribution to heads distance change; time spent face to face; time spent gazing at the game; overlap ratio; pause ratio; synchrony ratios).

Figure 1 summarizes how 2D+3D motion interaction features were obtained (computational details are given in ref. 25). (1) Play sessions took place in a consultation room. The parent and infant were invited to sit around a small table and to ‘to imagine you are having a tea party and play as you would do at home' for 4 min. (2) Two synchronized RGB-D sensors (Kinects) were placed in front of each participant and connected to a computer. This will run an acquisition application to record scene data. In addition, a camera was used to film the scene for the CIB evaluation. (3) To compute the projection matrix between the two Kinects to transform 3D points tracked by each Kinect into the same spatial/temporal basis, we performed a spatial calibration with a black and white chessboard and a temporal synchronization from the microphone outputs with hands clap. (4) The data captured by the Kinects were recorded for offline processing. For each sensor, saved data included a colour stream in an .avi video file (XVID codec) + timestamp for each image in an .xml file; a depth stream in an .avi video file (XVID codec) + timestamp for each image in an .xml file; and skeleton tracked points (position and orientation) in an .xml file. (5) The IMI2S computational framework^33^ was used to pre-process 3D skeleton data and, eventually, to extract behavioural features as described in ref. 25. From 2D+3D data and 3D space reconstruction, we extracted individual parameters (quantity of movement and Motion activity ratio for each partner) and dyadic parameters related to the dynamics of partners heads distance (contribution to heads distance), to the focus of mutual engagement (percentage of time spent face to face or oriented to the task), and to the dynamics of motion activity (synchrony ratio, overlap ratio, pause ratio). Definition of the 2D and 3D motion features are given in Table 2 together with illustrations and Information and Communication Technologies requirements. A video demo is also available as Supplementary Material Online.

### Statistical analysis and classification computing

The data for the present study were analysed using the statistical programme R, version 2.12.2 (R Foundation for Statistical Computing), with two-tailed tests and a 95% confidence level. Given the sample size for comparison of demographic and clinical characteristics, we used nonparametric Wilcoxon or Fisher tests. To assess how CIB composite scores and individual and dyadic motion features were related, we used Spearman's correlation coefficient. Given the sample size and the use of multiple statistics on the same data set, we used Holm correction to settle statistical significance. To compare control dyads versus dyads with mother showing neglect in the individual and dyadic motion features extracted during early interaction, we used binary classifiers. The classification results were obtained with an SVM (Support Vector Machine) classifier (linear kernel) and a 15 Cross-Validation approach. The SVM classification was performed using the R library ‘e1071'.

## Results

**Figure 2:**
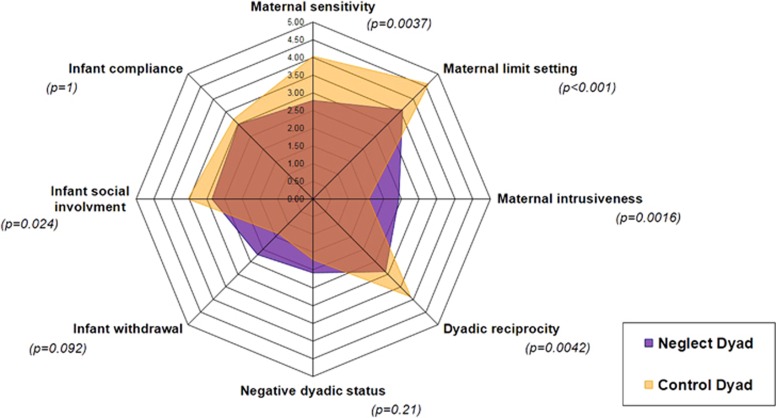
Radar diagram of the coding interactive behavior (CIB) during mother–infant early interaction according to groups: control dyads vs dyads with neglect mothers. With the CIB, better interaction is associated with higher scores in mother reciprocity and mother limit setting, infant compliance and infant social involvement, and dyadic reciprocity; as well as lower score in mother intrusiveness, infant withdrawal and dyadic negative status. Comparison between groups were done using Wilcoxon nonparametric test.

As expected, we found different CIB composite scores between control dyads and dyads with mothers showing neglect according to CIB mother and dyadic composite scores. Mean CIB results are summarized in Figure 2. Control dyads rated higher in CIB mother sensitivity and limit setting as well as in dyadic reciprocity. Also, they rated less in CIB mother intrusiveness. The CIB infant social engagement was better in control dyads. To assess the validity of the aforementioned 2D and 3D video motion features, we explored how they correlated CIB composite scores blindly rated from the same videos. Supplementary Table S1 (Supplementary Online) shows Spearman correlations between the mean values of the 2D and 3D motion features and CIB composite scores. We found several significant correlations with Spearman coefficients above 0.5. Interestingly, we found that maternal sensitivity and mother limit setting CIB scores had close and significant Spearman coefficients with mother quantity of movement, mother activity ratio, infant activity ratio, percentage of time spent face to face, synchrony ratio: parent response to infant, overlap ratio and pause ratio. In contrast, mother intrusiveness CIB score was significantly correlated with the same features (mother quantity of movement, mother activity ratio, infant quantity of movement, synchrony ratio: parent response to infant, overlap ratio and pause ratio) but in an opposite manner with the exception of pause ratio that had a different correlation profile with mother limit setting and mother intrusiveness CIB scores (see below and Supplementary Table S1, green boxes). Regarding dyadic CIB composite scores, we also found an oppositional profile between Dyadic reciprocity and negative dyadic status. Dyadic reciprocity was correlated with mother activity ratio, infant activity ratio, percentage of time spent face to face, synchrony ratio: parent response to infant, overlap ratio, and pause ratio. Negative dyadic status CIB score was significantly correlated with the same features but in an opposite manner (Supplementary Table S1, yellow boxes). Regarding the three infant-related CIB composite scores, most of the significant correlations were found with infant avoidance (Supplementary Table S1, pink boxes).

In the context of our experimental setup and considering only correlations remaining significant after Holm corrections, we found that mother motion activity ratio was negatively correlated with maternal sensitivity CIB score (*ρ*=−0.6, Holm corrected *P*=0.048) and with mother limit setting CIB score (*ρ*=−0.65, Holm corrected *P*=0.02) meaning that the less mothers showed motion activity during the task, the better was rated their sensitivity and their way to limit their child. In contrast, mother motion activity ratio was positively correlated with maternal intrusiveness CIB score (*ρ*=0.59, Holm corrected *P*=0.048) meaning that the more mothers showed motion activity during the task, the more they were judged as intrusive. The mother motion activity ratio was also positively correlated with infant avoidance CIB score (*ρ*=0.59, Holm corrected *P*=0.049) meaning that the more mothers showed motion activity, the more was the infant rated as withdrawn. The percentage of time spent face to face was negatively correlated with infant avoidance CIB score (*ρ*=−0.61, Holm corrected *P*=0.041) meaning that the less dyad spent time face to face, the more was the infant rated as withdrawn. Synchrony ratio: parent response to infant was negatively correlated with mother limit setting CIB score (*ρ*=−0.62, Holm corrected *P*=0.034) meaning that the less mothers showed motion response to the infant motion activity during the task, the better was rated their way to limit their child. In contrast, synchrony ratio: parent response to infant was positively correlated with maternal intrusiveness CIB score (*ρ*=0.61, Holm corrected *P*=0.038) and infant avoidance CIB score (*ρ*=0.59, Holm corrected *P*=0.045) meaning that the more mothers showed motion response to the infant during the task, the more they were judged as intrusive and the more the infant was rated as withdrawn. Overlap ratio tended to be significantly and positively correlated with infant avoidance CIB score (*ρ*=0.6, Holm corrected *P*=0.054) meaning that the more infants and mothers moved simultaneously during the task, the more the infant was rated as withdrawn. Finally, pause ratio was the motion feature that had the most meaningful correlations with CIB composite scores as seven out eight significant correlations were found. Pause ratio was negatively correlated with maternal sensitivity CIB score (*ρ*=−0.66, Holm corrected *P*=0.017), maternal intrusiveness CIB score (*ρ*=−0.63, Holm corrected *P*=0.02), negative dyadic status CIB score (*ρ*=−0.55, Holm corrected *P*=0.042) and with infant avoidance CIB score (*ρ*=−0.63, Holm corrected *P*=0.02) meaning that the less infant and mother kept not moving together during the task, the more the mother was rated sensitive or intrusive, the dyad rated in a negative status and the infant rated withdrawn. In contrast, pause ratio was positively correlated with mother limit setting CIB score (*ρ*=0.64, Holm corrected *P*=0.02), Dyadic reciprocity CIB score (*ρ*=0.61, Holm corrected *P*=0.021), and infant engagement CIB score (*ρ*=0.52, Holm corrected *P*=0.045), meaning that the more infant and mother kept not moving together, the better was rated mother way to limit her child, dyadic reciprocity and infant engagement.

To assess whether the aforementioned 2D+3D motion features brought enough information regarding the quality of early infant interaction, we performed a machine learning classification according to groups: control dyads vs dyads with mother showing neglect. Using Support Vector Machine (Linear Kernel) with two classes (each group) and 17 features (mean, standard deviation or ratio of the individual and dyadic 2D+3D motion features), we obtained an excellent classification with 100% of the cases correctly classified. A cross-validation approach was used to estimate the accuracy of the model. The data set was randomized before building splits, then each split served as a validation set on the model built on the remaining splits. After a 15-fold cross-validation, the total accuracy was 0.74 showing a good accuracy of the model.

## Discussion

The current results show that individual and dyadic motion features are highly correlated with CIB composite scores, which are coded globally by expert raters. In the context of a specific task in which mother and infant were asked to play at having a tea party on a small table, most of the correlation makes sense. On the basis of 2D+3D motion feature correlation profiles, we found that CIB maternal sensitivity and CIB mother limit setting composite scores were very similar, whereas both opposed to CIB mother intrusiveness composite score. In addition, we found an oppositional profile between CIB dyadic reciprocity and CIB negative dyadic status composite scores that fits well with the theoretical construct behind both dyadic dimensions of the CIB. Also, considering that half of the mothers had neglect behaviours, we are not surprised to find strong correlations with all three maternal CIB composite scores and both dyadic CIB composite scores. Finally, when we applied machine learning techniques using all aforementioned 2D–3D motion individual and dyadic motion features, we were able to classify 100% of the dyads in their corresponding group (control dyads vs dyads with mother showing neglect). This means that non-verbal motion behaviours are sufficient to classify and distinguish mothers showing neglect in contrast to control mothers. This further underlines the importance of synchrony and non-verbal features in social interaction.^15^

We believe the method developed here may have several implications and may open a new era in understanding interaction that we propose to call interaction imaging. First, it allows a finer understanding of interactions by changing the time scale (from a summary of the whole interaction with CIB towards a more fine-grained scale of the temporal flow) and by providing automatic quantitative features for the dynamics at individual and dyadic levels. This may have interesting applications in studies testing social interaction even if each scenario would have to select the pertinent parameters to be monitored as it is the case in imaging. Second, the current algorithms offer may help to develop automatic quantification of standardized assessments in the clinical setting. For example, the Autism Diagnostic Observation Schedule is a time-consuming assessment that requires specific expertise for rating.^35^ We suggest that interaction imaging might be of interest for providing automatic scoring if the assessment is recorded in an experimental room with the same sensors. Coupling with audio data would be warranted as ADOS includes language/communication assessment as well. Specific algorithm on audio speech turns are already available.^8^ Third, by offering interaction quantitative parameters, the current method may help investigating more deeply, the biology of interaction whether it is related to early interaction, conflict interaction and stressful interaction. Timely biological parameters are already available such as hormones (for example, oxytocin), peptides (for example, BDNF), physiological signals (for example, respiratory sinus arrhythmia) and brain activities (for example, qEEG)^13, 14^ and could be used in future studies combining both interaction and biological features. Finally, the same methods that we applied here to study early mother/infant interaction could be used to investigate motion quantitative parameters in the context of psychotherapeutic sessions. A recent preliminary study has already offered interesting insights by showing that coordinated body movement reflects relationship quality and outcome.^36^

The current methods still have limitations. First, motion features are extracted after 3D reconstruction that requires post processing. More computational development is needed to have an online fully automatized method. Second, regarding our setting, the interactive situation we proposed (the tea party) might have been difficult for young toddlers although validation starts at 12 months.^26^ However, they were only five participants aged younger than 18 months (two in the neglect group and three in the control group). Third, despite the cross-validation, the sample size remains a problem in terms of generalization of the SVM results: ideally the model should be tested on another sample, or the sample size should be increased to split it into a training set and a testing set.

We conclude that the proposed method may present a promising, low-cost methodology that can uniquely use artificial technology to detect meaningful features of human interactions and may have several implications for studying dyadic behaviours and interactive dynamics in psychiatry.

## Figures and Tables

**Table 1:** Demographics and clinical characteristics of the participants

	*Mothers showing neglect (*N*=10)*	*Healthy mothers (*N*=10)*	*Test*^a^	P
Mother age: mean (±s.d.) years	32.7 (±3.9)	34.8 (±5.1)	W=62	0.18
Infant age: mean (±s.d.) months	26.2 (±9.4)	23.2 (±7.7)	W=36.5	0.51
Infant sex: % of male (*N*)	60% (6 males, 4 females)	40% (4 males, 6 females)	Fisher^b^	0.82
DC 0–3 PIRGAS score: mean (±s.d.)	38.7 (±14.6)	78.6 (±10.2)	W=87	<0.001

Abbreviation: DC 0–3 PIRGAS, Parent–Infant Relationship Global Assessment Scale from the diagnostic classification 0 to 3.

a Wilcoxon test unless specified.

b Odds ratio=1.81; 95% confidence interval (0.22–16.64).

**Table 2:**
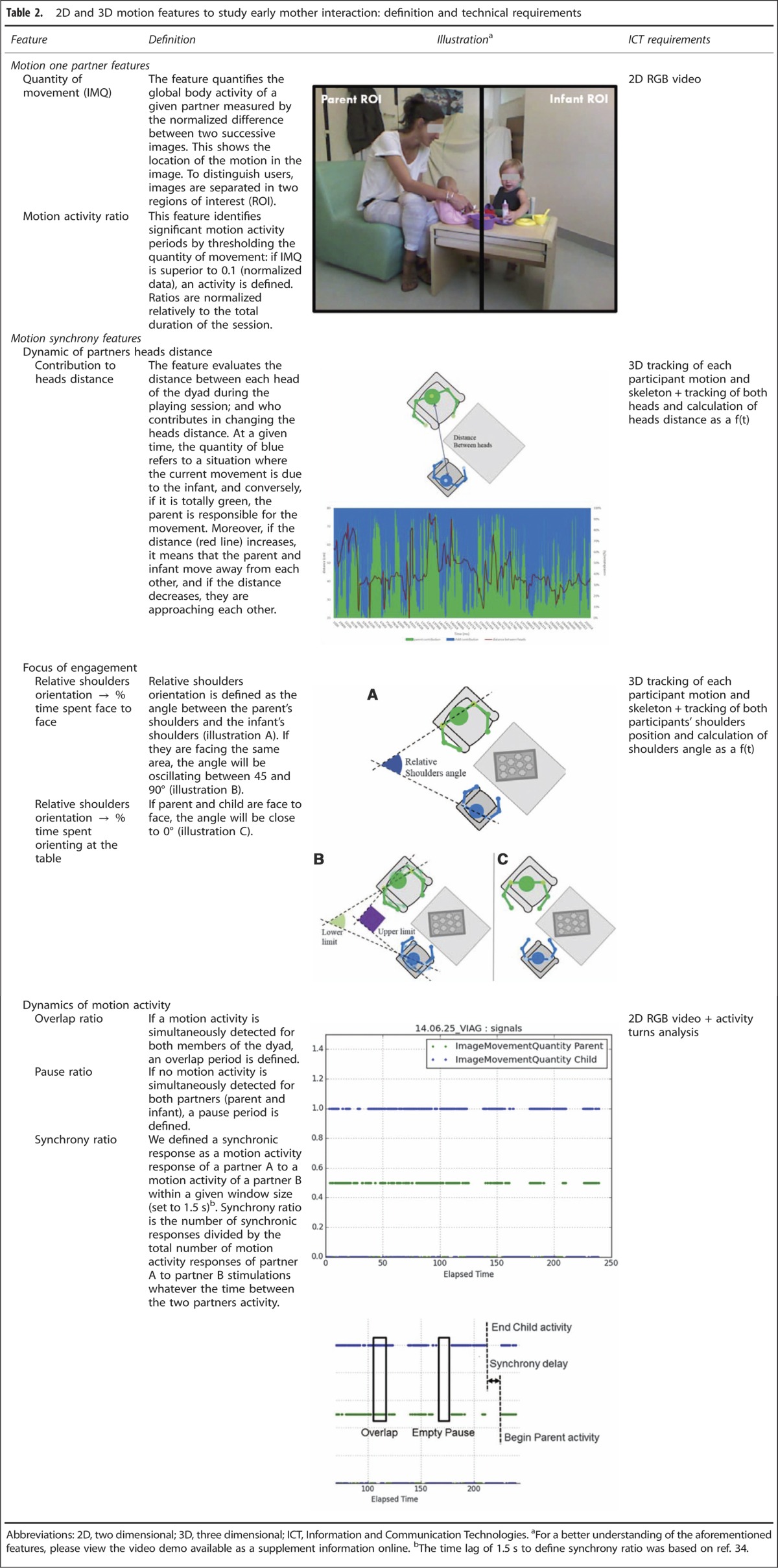
2D and 3D motion features to study early mother interaction: definition and technical requirements

## Supplementary Material

Supplementary Informations
