# Supplementary material for: Interaction and behaviour imaging: a novel method to measure mother–infant interaction using video 3D reconstruction
Source: Transl Psychiatry. 2016 May 24;6(5):e816–. doi: 10.1038/tp.2016.82 (PMC5070050; doi:10.1038/tp.2016.82)
Supplement: Supplementary Table 1 [file tp201682x2.pdf]

**Table S1. Correlation between CIB interactive composite scores and 2D and 3D motion features**								
	CIB interactive composites							
	**Maternal sensitivity**	**Mother limit setting**	**Mother intrusiveness**	**Dyadic reciprocity**	**Negative dyadic status**	**Infant avoidance**	**Infant engagement**	**Infant compliance**
Individual parameters								
Mother quantity of movement	-0.44 *p=0.06*	**-0.54** ***p=0.018***	**0.58** ***p=0.009***	-0.44 *p=0.06*	0.15 *p=0.53*	0.22 *p=0.37*	-0.44 *p=0.06*	-0.35 *p=0.14*
Mother activity ratio	**-0.6** ***p=0.007****	**-0.65** ***p=0.003****	**0.59** ***p=0.008****	**-0.53** ***p=0.02***	**0.47** ***p=0.044***	**0.59** ***p=0.008****	-0.44 *p=0.06*	-0.24 *p=0.32*
Infant quantity of movement	-0.26 *p=0.28*	-0.35 *p=0.147*	**0.48** ***p=0.038***	-0.27 *p=0.26*	-0.08 *p=0.74*	0.15 *p=0.54*	-0.2 *p=0.41*	-0.05 *p=0.84*
Infant activity ratio	**-0.48** ***p=0.037***	-0.36 *p=0.13*	0.31 *p=0.19*	**-0.47** ***p=0.04***	0.4 *p=0.088*	**0.47** ***p=0.044***	-0.37 *p=0.12*	-0.36 *p=0.13*
Synchrony parameters								
Dynamics of partners heads distance								
Mother contribution to heads distance	-0.09 *p=0.71*	0.23 *p=0.34*	0.18 *p=0.47*	-0.14 *p=0.56*	-0.21 *p=0.39*	-0.28 *p=0.25*	-0.12 *p=0.63*	0.34 *p=0.15*
Infant contribution to heads distance	0.1 *p=0.67*	-0.22 *p=0.37*	-0.19 *p=0.43*	0.16 *p=0.52*	0.2 *p=0.42*	0.26 *p=0.28*	0.14 *p=0.58*	-0.33 *p=0.17*
Focus of engagement								
% of time spent face to face	0.41 *p=0.08*	0.51 *p=0.026*	-0.34 *p=0.15*	0.44 *p=0.057*	-0.44 *p=0.057*	**-0.61** ***p=0.005****	0.42 *p=0.076*	0.37 *p=0.12*
% of time spent looking together at the table	-0.2 *p=0.4*	-0.14 *p=0.57*	0.06 *p=0.8*	-0.25 *p=0.29*	-0.06 *p=0.81*	0.03 *p=0.9*	-0.26 *p=0.275*	-0.13 *p=0.59*
Dynamics of motion activity								
Synchrony ratio: Parent response to Infant	**-0.54** ***p=0.017***	**-0.62** ***p=0.004****	**0.61** ***p=0.005****	**-0.48** ***p=0.039***	**0.48** ***p=0.038***	**0.59** ***p=0.008****	-0.41 *p=0.08*	-0.22 *p=0.38*
Synchrony ratio: Infant response to Parent	-0.2 *p=0.4*	-0.12 *p=0.62*	0.09 *p=0.7*	-0.23 *p=0.34*	0.22 *p=0.37*	0.28 *p=0.24*	-0.26 *p=0.28*	**-0.46** ***p=0.047***
Overlap ratio	**-0.56** ***p=0.012***	**-0.53** ***p=0.02***	**0.46** ***p=0.049***	**-0.5** ***p=0.03***	**0.5** ***p=0.028***	**0.6** ***p=0.007****	-0.38 *p=0.11*	-0.18 *p=0.46*
Pause ratio	**-0.66** ***p=0.002****	**0.64** ***p=0.003****	**-0.63** ***p=0.004****	**0.61** ***p=0.005****	**-0.55** ***p=0.014****	**-0.63** ***p=0.004****	**0.52** ***p=0.023****	0.44 *p=0.06*

CIB=Coding Interactive Behavior; *p≤0.05 with Holm corrected p-value.
